# Plasticity of Blood Corpuscles

**Published:** 1864-01

**Authors:** 


PLASTICITY OF BLOOD CORPUSCLES.—Dr. Sharpey says: "The plasticity of the blood corpuscle is unrivaled by any other physical body. It will assume all sorts of protean shapes under the slightest influences. Elongating to a mere thread, it will pass through a narrow chink; it will wrap itself round an accute projecting angle, or protrude feelers and tails under the influence of currents. In its natural state, it possesses sufficient elasticity to resume its original shape on the cessation of the modifying influences; but when gum or gelatine has been added, or when the plasma has been permitted to thicken spontaneously, the corpuscle retains any form it may have assumed till again altered by fresh influence."—*Proceedings of the Royal Society.—Intellectual Observer.*

